# Identification of QTL for perenniality and floral scent in cowpea (*Vigna unguiculata* [L.] Walp.)

**DOI:** 10.1371/journal.pone.0229167

**Published:** 2020-04-28

**Authors:** Sassoum Lo, Christian Fatokun, Ousmane Boukar, Paul Gepts, Timothy J. Close, María Muñoz-Amatriaín

**Affiliations:** 1 Department of Botany and Plant Sciences, University of California Riverside, Riverside, CA, United States of America; 2 International Institute of Tropical Agriculture, Ibadan, Nigeria; 3 Department of Plant Sciences, University of California Davis, Davis, CA, United States of America; 4 Department of Soil and Crop Sciences, Colorado State University, Fort Collins, CO, United States of America; Washington University, UNITED STATES

## Abstract

Perennial habit and floral scent are major traits that distinguish domesticated cowpeas from their wild relatives. However, the genetic basis of these two important traits remains largely unknown in cowpea. Plant longevity, a perenniality-related trait, and floral scent, an outcrossing trait, were investigated using a RIL population derived from a cross between a domesticated and a wild cowpea. QTL analysis revealed three significant loci, one on chromosome 8 associated with plant longevity and two, on chromosomes 1 and 11, for floral scent. Genes within the QTL regions were identified. Genes encoding an F-box protein (*Vigun08g215300*) and two kinases (*Vigun08g217000*, *Vigun08g217800*), and involved in physiological processes including regulation of flowering time and plant longevity, were identified within the perenniality QTL region. A cluster of O-methyltransferase genes (*Vigun11g096800*, *Vigun11g096900*, *Vigun11g097000*, *Vigun11g097600*, and *Vigun11g097800*) was identified within the floral scent QTL region. These O-methyltransferase cowpea genes are orthologs of the Arabidopsis *N*-*acetylserotonin O*-*methyltransferase* (*ASMT*) gene, which is involved in the biosynthesis of melatonin. Melatonin is an indole derivative, which is an essential molecule for plant interactions with pollinators. These findings lay the foundation for further exploration of the genetic mechanisms of perenniality and floral scent in cowpea. Knowledge from this study can help in the development of new extended-growth cycle lines with increased yield or lines with increased outcrossing for population breeding.

## Introduction

Cowpea (*Vigna unguiculata* [L.] Walp.) is a warm season legume of major importance for worldwide food and nutritional security. It is an annual, diploid (2n = 22) species with a morphologically and genetically diverse gene-pool composed of cultivated forms and several wild taxa [1]. Cowpea was domesticated in Africa [2, 3], from where it spread to other continents. Although there is a lack of consensus on where in Africa cowpea domestication occurred, it is believed that *V*. *unguiculata* subsp. *dekindtiana* is the probable wild progenitor of cultivated cowpea [4, 5]. The main characteristics of *V*. *unguiculata* subsp. *dekindtiana* include perenniality, hairinesss, and small size of pods and seeds [6]. Although this wild gene-pool could be a potential source of favorable alleles for traits related to resistance to biotic and abiotic stresses (e.g., aphid resistance, Striga resistance, and drought and heat tolerance), it has remained largely unexplored by breeders for the development of improved cultivars. Increased knowledge of the genomic regions controlling domestication-related traits (DRTs) is needed to exploit cowpea wild germplasm efficiently.

Cowpea domestication involved considerable phenotypic changes from subsp. *dekindtiana*, including reduction of pod shattering, increased size of edible organs, loss of perenniality, and changes in flower color and scent. Compared to many other crop species, only a few studies have identified loci associated with DRTs in cowpea. Those studies made use of bi-parental populations of recombinant inbred lines (RILs) derived from a cross between a domesticated and a wild cowpea [7–9]. In particular, Andargie et al. [7] and Lo et al. [9] identified QTLs for several DRTs including ovule number, seed germination, seed coat thickness, pod shattering and days to flowering, while the study of Fatokun et al. [8] focused on seed size. However, the genetics of other important DRTs such as perenniality and flower scent remains unknown.

Developing perennial crops has been a longstanding goal in many breeding programs, especially those of cereal crops [10]. Perennial grain crops have been proposed as a strategy to address agriculture’s global challenges such as land degradation, food insecurity and climate change [10]. Perennial species have longer growing periods leading to increased photosynthate assimilation, and extensive root systems, which help decrease nitrate runoff, increase soil sequestration of carbon and decrease soil erosion [11]. Furthermore, perennial crops are reported to be more resilient to abiotic stress resulting from weather fluctuations and may require less herbicide treatment [12]. In cowpea, developing perennial varieties could be beneficial for grain yields and the improvement of soil fertility. In addition, as cowpea fodder is highly favored by livestock farmers in the dry savanna regions of sub-Saharan Africa, perennial cowpea varieties could provide a reliable source of fodder for livestock by surviving the dry season. However, to our knowledge there have been no reports on efforts to introgress perenniality from wild cowpea into elite lines.

Genes associated with perenniality-related traits have been explored mostly in Arabidopsis. Several of these studies have identified genes known to regulate flowering time and meristem determinacy such as *SUPPRESSOR OF OVEREXPRESSION OF CONSTANS 1 (SOC1)*, *APETALA1(AP1)*, *FRUITFULL (FUL)* [13, 14]. Another gene, *TERMINAL FLOWER 1* (*TFL1*), has been suggested to play an important role in perennialism by controlling the juvenile to adult transition phase and contributing to the polycarpic growth habit [15, 16]. Also, orthologs of *TFL1* have been shown to be associated in polycarpic growth habit in other perennial species including ryegrass [17], apple [18], *Populus* sp. [19] and *Arabis alpina* [20]. In legumes, *TFL1* orthologs including *Dt1* in soybean [21], *PvTFL1y* in common bean [22], *CcTFL1* in pigeonpea [23] have been reported to control determinate growth habit.

Another domestication syndrome trait in cowpea is floral scent, which facilitates plant-pollinator interaction. Pollination has been reported to contribute to more than one third of crop yield [24] and could assist in population breeding and the development of F_1_ hybrid varieties. In addition, as cowpea varieties carrying a transgenic resistance gene (*Bt*) derived from *Bacillus thuringiensis* are being evaluated in some African countries, understanding the genetics of floral scent would help inform insect pollination behavior and flight distances [25] and environmental biosafety policy and regulation related to transgene scape.

Floral scent-related genes have been characterized from several model plants. Those mostly encode enzymes including Iso-eugenol O-methyltransferase, benzylalcohol acyltransferase, orcinol O-methyltransferase and phloroglucinol O-methyltransferase [26–29]. In cowpea, a previous study identified loci associated with floral scent compounds [30]. The authors identified QTLs influencing 23 volatile compounds including nitrogen compounds. Here we studied the genetic differences in scent production between wild and domesticated cowpea.

In the present study, we identified loci associated with perenniality and floral scent using a wild by cultivated RIL population [9], a high-density SNP genotyping array [31], a reference genome sequence [32] and new phenotypic data. Knowledge from this study can guide the development of cowpea perennial lines leading to an increase of cowpea grain and fodder productivities.

## Materials and methods

### Plant material and phenotypic data collection

A biparental mapping population of 170 recombinant inbred lines (RILs) derived from a cross between a wild and a domesticated cowpea accession and developed at the International Institute of Tropical Agriculture (IITA) was used to evaluate perenniality and floral scent. The development of this population is described in Lo et al. [9]. Briefly, the wild parent (TVNu-1158) is a small-seeded and aphid-resistant accession with a perennial growth habit and scented flowers. The cultivated parent (IT99K-573-1-1) is an early-maturing, white-seeded, high-yielding and Striga resistant variety with an annual life cycle and non-scented flowers. The parents and the RILs were sown on February 20^th^, 2017 in pots filled with 5.0 kg topsoil and placed in a screen house at IITA, Ibadan, Nigeria (latitude 7°30′N and longitude 3°54′E, elevation 240 masl). Five to eight seeds of each RIL were sown per pot. When seedlings were well established (as rate of growth initially varied between seedlings of the RILs) the number of plants was reduced to three per pot. The plants were thereafter allowed to grow until death was recorded on the last of the three plants. Perenniality was scored as the number of days from planting to when the plant died. Floral scent was scored as a qualitative trait by human olfaction on five newly opened flowers as “scented” (score = 1) or “non-scented” (score = 0).

### QTL analysis and identification of candidate genes

The RIL population was genotyped with the Cowpea iSelect Consortium Array, which includes 51,128 SNPs [31] (Muñoz‐Amatriaín et al., 2017). SNPs were called using the GenomeStudio software V.2011.1 (Illumina, Inc., San Diego, CA, USA). Data curation was performed by removing SNPs with more than 20% missing and/or heterozygous calls and minor allele frequencies <0.25. MSTmap [33] (http://www.mstmap.org/) was used for constructing the genetic map, which consisted of 17,739 SNPs mapped to 1,825 unique positions [9]. The chromosomes were numbered and oriented according to the cowpea pseudomolecules [32].

QTL mapping was performed using a linear mixed model (*y* = *Xβ+Zkak+Wkdk+ξ+ϵ*) [34] implemented in R as described in Lo et al. [9]. A genome-wide critical value was calculated with a modified Bonferroni correction and used as the threshold for the detection of significant QTL. The modified Bonferroni used the effective degrees of freedom of the trait as the denominator. The effective degrees of freedom was defined as $m0=\frac{\sum \left(Wk-1\right)}{Wk}$, where Wk is the Wald test statistic for SNP k. The trait specific Bonferroni corrected critical value was -log_10_ (0.05/m0). A SNP was declared as significant if its -log_10_ (*P*-value) was larger than -log_10_ (0.05/m0) [9]. The proportion of phenotypic variance contributed by each significant SNP was calculated using the following formula: $\frac{var(X)a^{2}}{var(y)}$, where X is a variable holding the genotype code (+1,-1) for SNP k, var(X) is the variance of variable X, a is the estimated effect for SNP k and var(y) is the total phenotypic variance of the trait under study. The effect of each marker was estimated as a fixed effect and tested using the Wald test statistic (squared effect divided by the variance of the estimated effect).

The physical region of the QTL was determined by positioning flanking SNPs in the reference genome sequence [32] and annotated gene models underlying the QTL region were identified.

## Results and discussion

### Phenotypic variation in the population

DRTs “perenniality” and “flower scent” were evaluated in this study. Phenotypic values obtained from the parents and the 170 lines in the population are reported in S1 Table. Perenniality (plant longevity) was scored as the number of days from planting to when the plant died. IT99K-573-1-1 has an annual life cycle and died 123 days after planting, while TVNu-1158 died 700 days after planting (Table 1). In the RIL population, the number of survived days ranged from 94 to >774 days (Table 1), and mean and standard deviation were calculated. Transgressive segregation was observed suggesting that more than one locus could contribute to plant longevity. The frequency distribution of plant longevity did not fit a normal distribution as it was skewed positively towards a longer life span (S1 Fig).

**Table 1:** Phenotypic values of the parental lines and the RIL population. NA: not applicable.

Trait	IT99K-573-1-1	TVNu-1158	RIL population	
			*Mean +/- SE*	*Range*
Plant longevity (#days)	123	700	325 +/- 142	94 - >774
Floral scent	non-scented	scented	NA	NA

Floral scent was scored qualitatively by human olfaction as scented and non-scented flowers. The domesticated parent IT99K-573-1-1 had non-scented flowers while TVNu-1158 had scented flowers. In the population, 51 RILs had scented flowers and 119 lines had non-scented flowers (Table 1). A chi square goodness-of-fit test suggests that the segregation pattern of floral scent in the RIL population did not fit a Mendelian 1:1 ratio (P-value = 1.83E-07).

### QTLs for perenniality on chromosome 8 and floral scent on chromosomes 1 and 11

**Fig 1:**
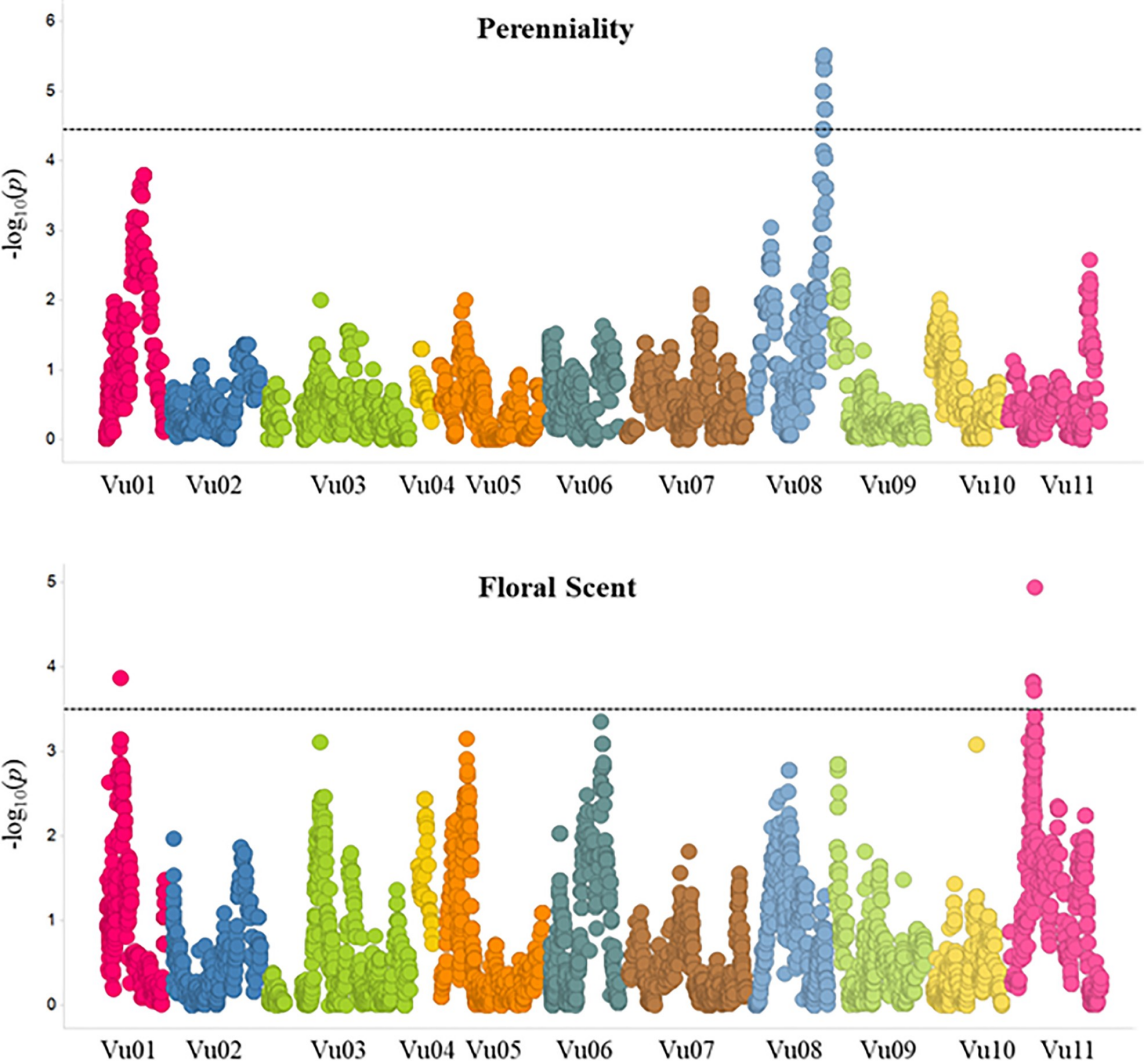
QTL plots for perenniality and floral scent. The horizontal axis indicates the chromosomes, the vertical axis indicates the -log_10_ of the probability (*P*-values). The dashed line indicates the significance threshold.

Using a mixed linear model for QTL analysis developed by [34], one QTL for perenniality was identified on chromosome 8 (Vu08) and 2 QTL were identified for floral scent on chromosomes 1 (Vu01) and 11 (Vu11) (Fig 1; Table 2). The effect of each QTL was determined and reported in Table 2. The effect of these QTLs might be artificially increase due to small population size.

**Table 2:** QTL for perenniality and flower scent identified by the linear mixed model analysis.

Trait	Peak SNP	Chr.	Position (cM)	Position (bp)	-Log10(P)	QTL region (cM)	% Phenotypic variation	Effect
Perenniality (Plant longevity)	2_14129	8	79.58	37725907	5.51	78.87–80.67	26.45	25.16
Flower scent	2_51494	1	14.74	27704236	3.87	14.74–15.03	9.5	-0.15
Flower scent	2_00068	11	27.4	27125507	4.94	26.81–28	13.75	-0.18

The QTL for perenniality explained 26% of the phenotypic variance (Table 2), spanned 527 kb (37,388,250 to 37,914,805 bp) on Vu08 and contained 79 gene models [32] (S2 Table). QTLs associated with perenniality-related traits have been identified mostly in cereal crops including maize [35], wheat [36] and rice [37]. In mungbean (*Vigna radiata*) the inheritance of perenniality through the presence or absence of tuberous roots was studied by Nguyen et al. [38]. Authors proposed that the formation of tuberous root (a perenniality-related trait) might be conditioned by two complementary dominant loci. However, these cited studies were interested in perennialism based on traits such as regrowth and formation of a rhizome, while the present study focuses on plant longevity. Our results reinforce the view that plant longevity is a component of perennialism [39]. The plant longevity locus could assist breeding programs in the development of perennial cowpea. In cereal crops, loci associated with perenniality-related traits have been used to confer perennial habit on annual elite lines. For example, Cox et al. [40] used loci associated with rhizome production to develop perennial lines derived from crosses between *Sorghum bicolor* and either *S*. *halepense* or *S*. *propinquum* with the goal of improving grain yields and seed weights and preventing and reversing soil degradation in the sorghum growing region.

Floral scent is an important trait to attract pollinators and could also be involved in repelling pests [41]. In the present study two QTLs associated with the emission of scented flowers were detected, one each on Vu01 and Vu11 (Fig 1). The QTL on Vu01 explained 9.5% of the variation of the trait while the QTL on Vu11 accounted for ~14% (Table 2). The significant region on Vu01 spanned 196 kb (27,704,236–27,900,691 bp) and contained 14 annotated genes, while the QTL on Vu11 spanned 6 Mb (23,241,654–29,117,703 bp) and contained 201 gene models [32] (S2 Table). A previous study in cowpea reported a total of 63 QTLs influencing 23 floral scent compounds [30]. Because of the unavailability of marker sequences used in that study, the possible overlap between the loci identified here and those from the Andargie et al. [30] could not be determined. In addition to mapping two main QTLs, the present study identified noteworthy candidate genes associated with floral scent. For the QTL on Vu11, the peak SNPs are 800 kb away from a cluster of O-methyltransferase family protein genes, which are involved in the biosynthetic process of aromatic compounds in plants [42].

### Identification of candidate genes

A total of 79 genes were identified within the QTL region for perenniality. Among these are a gene encoding histidine kinase (*Vigun08g217000*), a protein kinase superfamily protein (*Vigun08g217800*), and an F-box family protein gene (*Vigun08g215300*). Genes encoding two kinases and an F-box protein have been reported as candidates that potentially impact the life history switch from perennial to annual of different Arabidopsis species [43]. Furthermore, those authors suggested that the F-box gene is the best candidate for future functional studies based on the dN (nonsynonymous substitutions per nonsynonymous site) to dS (synonymous substitutions per synonymous site) ratio. In addition, F-box genes have been known to influence a variety of biological processes essential for plant growth and development [44]. The Arabidopsis ortholog of *Vigun08g215300* is *AT5G48170* (*SNEEZY*), which is a regulator of gibberellin (GA) signaling [45, 46]. *SNEEZY* mutations cause phenotypes resulting from reduced GA response including delayed flowering. Moreover, Ariizumi and Steber [46] reported that *SNEEZY* overexpression has an impact in apical dominance and growth habit. The other perenniality candidate is *Vigun08g217000* encoding a histidine kinase. *Vigun08g21700000* is located in the QTL peak region and its Arabidopsis ortholog *AHK2* has been shown to regulate plant longevity [47]. Gain-of-function mutation of *AHK2* increased plant longevity and prolonged the reproductive growth phase in Arabidopsis [47]. Interestingly, this gene has been identified as a candidate for other domestication-related traits in cowpea including seed size [32, 48]. The last candidate gene, *Vigun08g217800*, encodes a kinase superfamily protein and is an ortholog of the Arabidopsis gene *AT5G55560*, which is a serine/threonine-protein kinase member of the WNK gene family. Genes belonging to the WNK family are involved in various physiological processes including regulation of flowering time by modulating the photoperiod pathway [49, 50]. Further studies such as fine mapping, mutant analysis, and gene expression will be required to explore the role these candidate genes in the perennial to annual switch in cowpea.

**Fig 2:**
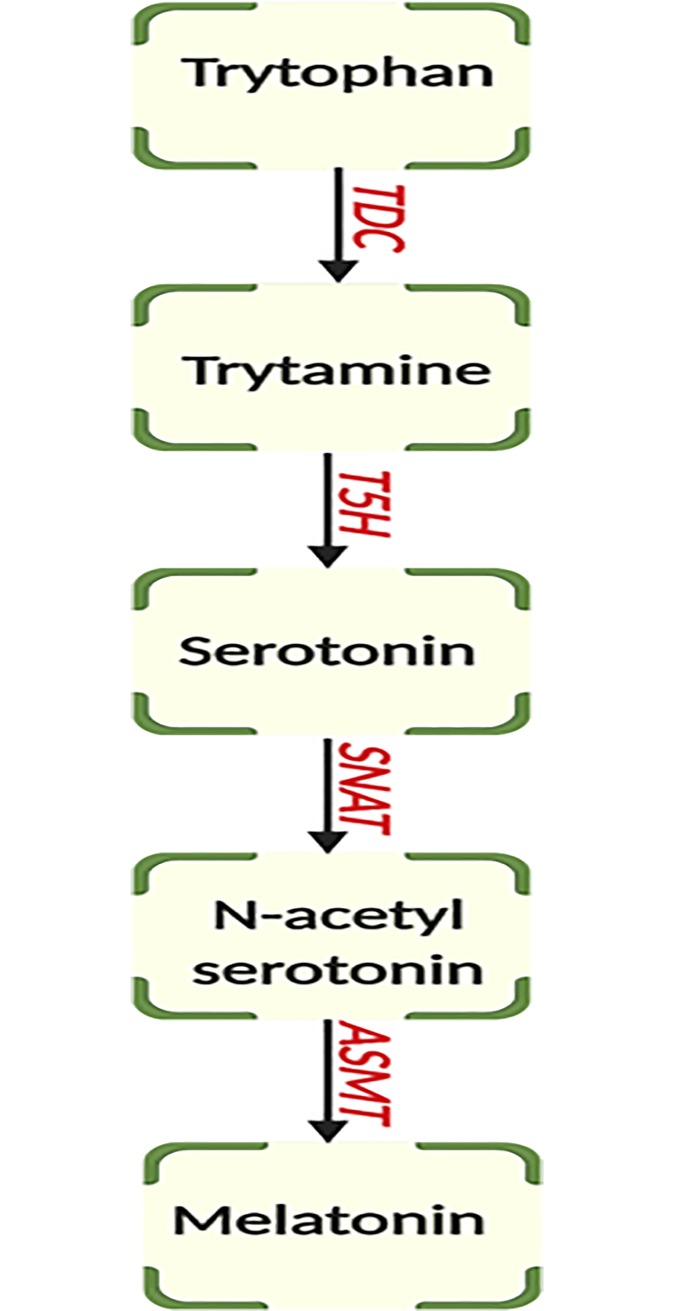
Schematic view of melatonin biosynthesis pathway. TDC: tryptophan decarboxylase, T5H: tryptamine 5-hydroxylase, SNAT: serotonin N-acetyltransferase, ASMT: N-acetylserotinin methyltransferase.

No obvious candidate genes were identified for the minor floral scent QTL on Vu01. However, O-methyltransferase family protein genes were found within the Vu11 QTL. Expression data from Yao et al. [51] available at legumeinfo.org showed that five of these genes (*Vigun11g096800*, *Vigun11g096900*, *Vigun11g097000*, *Vigun11g097600* and *Vigun11g097800*) were expressed in flower tissues (S2 Fig). These genes are orthologs of the Arabidopsis gene *AT4G35160* (*ASMT*), which encodes a cytosolic N-acetylserotonin O-methyltransferase. *ASMT*, together with other genes such as *tryptophan decarboxylase* (*TDC*), *tryptamine 5-hydroxylase* (*T5H*) and *serotonin N-acyltransferase* (*SNAT*), have been implicated in the process of melatonin synthesis from tryptophan [52–54]. Fig 2 shows a diagram of one of the four pathways proposed in the biosynthesis of melatonin with *ASMT* responsible for the transformation of N-acetylserotonin to melatonin. Melatonin (N-acetyl-5 methoxytryptamine) is an indole derivative (with an indole nucleus). Indole is one of the major metabolites of scent [55]. In addition, indole is one of the several volatiles that attract pollinators and contribute to defense [56, 57]. Indole is one of the floral scent compounds identified in cowpea [30], which was also associated with two QTLs in that study. Thus, these O-methyltransferase genes are promising candidates to study the genetic mechanism of floral scent in cowpea.

This study provides a step towards understanding the genetic architecture of perenniality and floral scent in cowpea. Deciphering the genetics of these two DRTs would help efforts to perennialize domesticated cowpea, domesticate new crops from wild cowpea and develop new varieties with the aim of increasing yield and other quality traits.

## Supporting information

S1 Fig Phenotypic distribution of perenniality and floral scent. (TIF)

S2 Fig Expression data of the floral scent candidate genes. TPM: Transcripts Per Million; dap: days after pollination. Data from Yao et al (2016) and available at legumeinfo.org.
(TIF)

S1 Table (XLSX)

S2 Table (XLSX)
