# Supplementary figures and images for: Identification of QTL for perenniality and floral scent in cowpea (Vigna unguiculata [L.] Walp.)
Source: PLoS One. 2020 Apr 28;15(4):e0229167. doi: 10.1371/journal.pone.0229167 (PMC7188242; doi:10.1371/journal.pone.0229167)

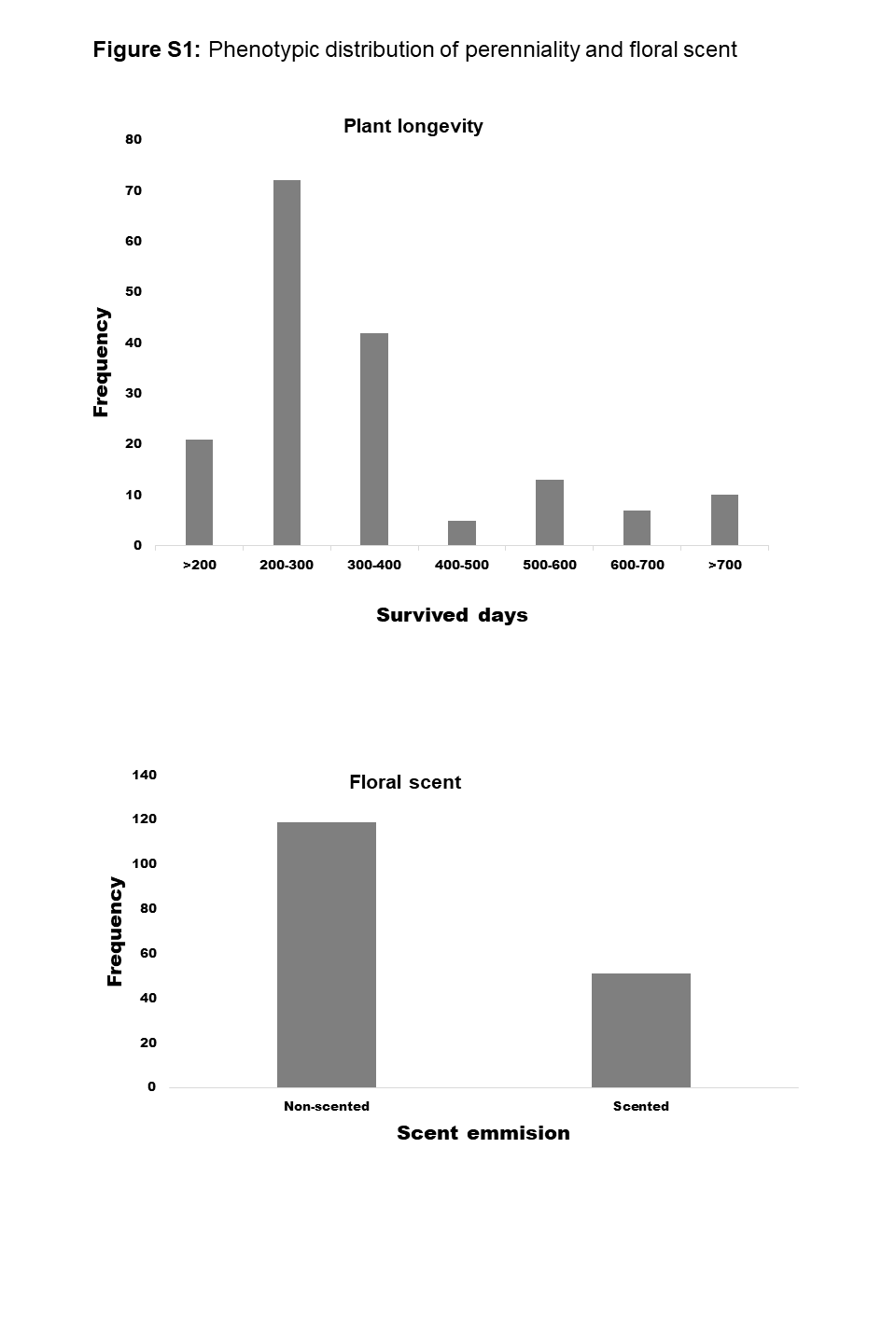

Supplement: S1 Fig — (TIF) [file pone.0229167.s001.tif]

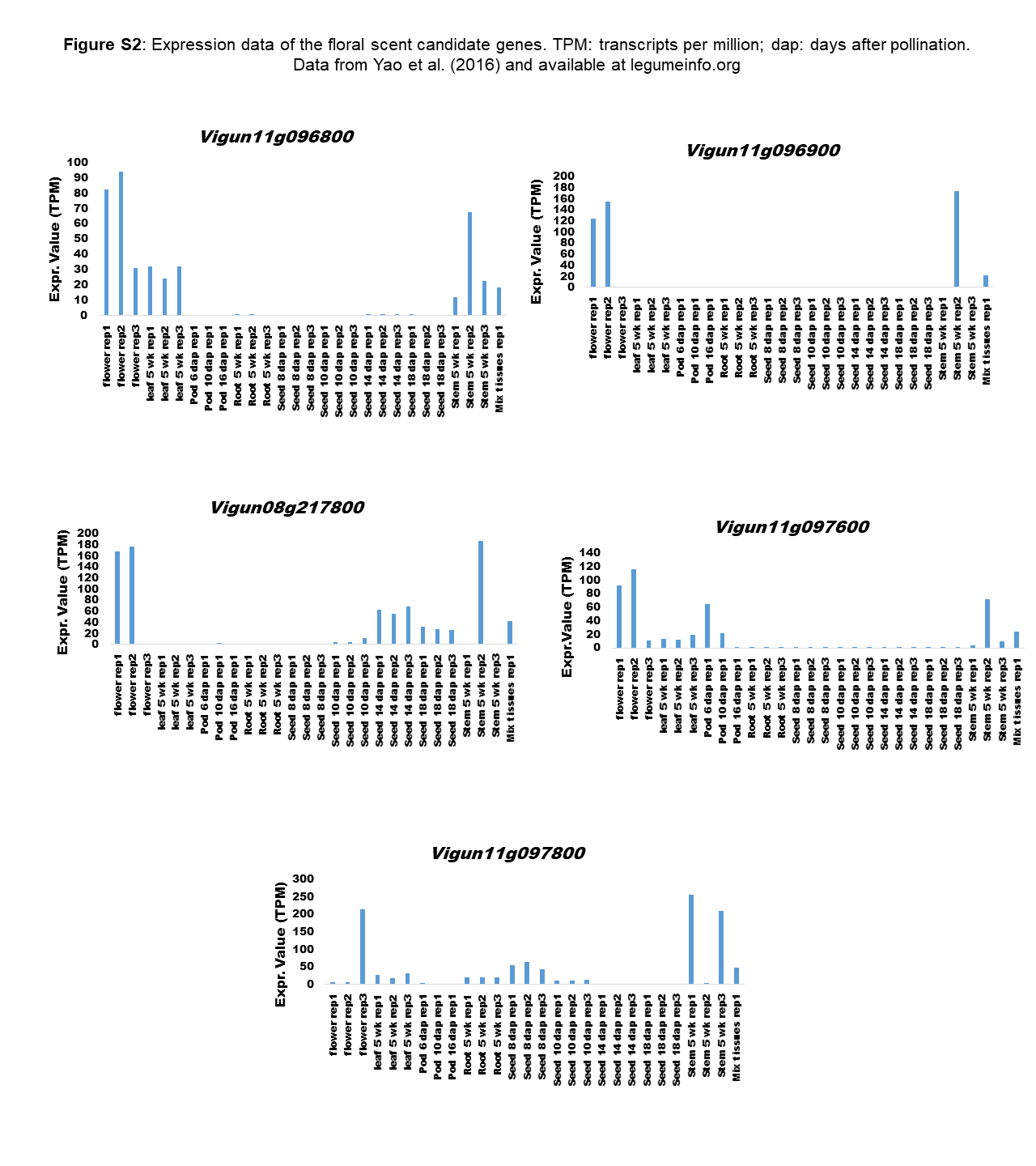

Supplement: S2 Fig — TPM: Transcripts Per Million; dap: days after pollination. Data from Yao et al (2016) and available at legumeinfo.org. (TIF) [file pone.0229167.s002.tif]
